# Defining Lifetime Risk Thresholds for Breast Cancer Surgical Prevention

**DOI:** 10.1001/jamaoncol.2025.2203

**Published:** 2025-07-24

**Authors:** Xia Wei, Lea Mansour, Samuel Oxley, Caitlin T. Fierheller, Ashwin Kalra, Jacqueline Sia, Subhasheenee Ganesan, Michail Sideris, Li Sun, Adam Brentnall, Stephen Duffy, D. Gareth Evans, Li Yang, Rosa Legood, Ranjit Manchanda

**Affiliations:** 1 Department of Health Services Research and Policy, London School of Hygiene & Tropical Medicine, London, United Kingdom; 2 Wolfson Institute of Population Health, Queen Mary University of London, London, United Kingdom; 3 Department of Gynaecological Oncology, Barts Health NHS Trust, Royal London Hospital, London, United Kingdom; 4 Manchester Centre for Genomic Medicine, Division of Evolution, Infection and Genomic Sciences, University of Manchester, MAHSC, Saint Mary’s Hospital, Manchester, United Kingdom; 5 School of Public Health, Peking University, Beijing, China

## Abstract

**Importance:**

Expanding access to genetic testing and availability of validated breast cancer (BC) risk prediction models are increasingly identifying women at elevated BC risk who do not carry high-penetrance *BRCA1*/*BRCA2*/*PALB2* pathogenic variants. The precise BC risk threshold for offering risk-reducing mastectomy (RRM) for BC prevention is unknown.

**Objective:**

To define the lifetime BC risk thresholds for RRM to be cost-effective compared with nonsurgical alternatives for BC prevention.

**Design, Setting, and Participants:**

This economic evaluation used a decision-analytic Markov model to compare the cost-effectiveness of RRM with BC screening and medical prevention in a simulated cohort. Extensive sensitivity analyses were performed. The study setting was from a UK payer perspective over a lifetime horizon until age 80 years. The simulated cohort included women aged 30 to 60 years at varying lifetime BC risks from 17% to 50%. The study was conducted between September 2022 and September 2024.

**Exposures:**

Undergoing RRM or receiving risk-stratified BC screening with medical prevention (tamoxifen or anastrozole).

**Main Outcomes and Measures:**

The incremental cost-effectiveness ratio was calculated as incremental cost per quality-adjusted life-year (QALY) gained and compared with the UK willingness-to-pay (WTP) threshold of £20 000 (US $27 037) to £30 000 (US $40 555) per QALY. BC cases prevented were estimated at the population level.

**Results:**

In the simulated cohort of 100 000 thirty-year-old women in the UK, undergoing RRM became cost-effective at a 34% lifetime BC risk using the £30 000 (US $40 555) per QALY WTP threshold. This increased to a 42% lifetime BC risk using the £20 000 (US $27 037) per QALY WTP threshold. The identified lifetime BC risk thresholds for RRM to be cost-effective among women aged 35, 40, 45, 50, 55, and 60 years were 31%, 29%, 29%, 32%, 36%, and 42%, respectively, using the £30 000 (US $40 555) per QALY WTP threshold. Overall, undergoing RRM was deemed cost-effective for women aged 30 to 55 years with a lifetime BC risk of at least 35%, with more than 50% of simulations being cost-effective in probabilistic sensitivity analysis. Offering RRM for women with a lifetime BC risk of 35% or higher could potentially prevent approximately 6538 (95% CI, 4454-7041), or approximately 11% (95% CI, 8%-12%), of the 58 756 BC cases occurring annually in women in the UK. In the probabilistic sensitivity analysis, 20.71% to 59.96%, 44.04% to 81.29%, and 97.26% to 99.35% of simulations were cost-effective for women with 35%, 40%, and 50% lifetime BC-risk undergoing RRM at age 30 under the £20 000 to £30 000 per QALY WTP threshold, respectively.

**Conclusions and Relevance:**

In this economic evaluation, undergoing RRM appears cost-effective for women aged 30 to 55 years with a lifetime BC risk of 35% or higher. These results could have significant clinical implications to expand access to RRM beyond *BRCA1/BRCA2/PALB2* pathogenic variant carriers. Future studies evaluating the acceptability, uptake, and long-term outcomes of RRM among these women are warranted.

## Key Points

**Question:**

At what level of lifetime breast cancer risk is offering risk-reducing mastectomy cost-effective compared with nonsurgical alternatives for breast cancer prevention?

**Findings:**

In this economic evaluation of a simulated cohort of women at varying risks for breast cancer, undergoing risk-reducing mastectomy was found to be cost-effective for women in the UK aged 30 to 55 years with a lifetime breast cancer risk greater than 35%, compared with risk-stratified breast cancer screening and medical prevention (tamoxifen or anastrozole).

**Meaning:**

These findings support changing current practice to expand risk-reducing mastectomy access beyond the traditional *BRCA1*, *BRCA2*, and *PALB2* pathogenic variant carriers to individuals at a 35% or higher lifetime risk.

## Teaser

This economic evaluation defines the lifetime breast cancer risk thresholds for risk-reducing mastectomy to be cost-effective compared with nonsurgical alternatives for breast cancer prevention.

## Introduction

Validated personalized risk prediction models incorporating genetic (cancer susceptibility gene [CSG] and polygenic risk score [PRS]) and nongenetic (family history [FH]/epidemiologic/reproductive/hormonal profile/mammographic density) factors are available and increasingly used to identify women at elevated breast cancer (BC) risk.^1,2,3,4^ Genetic testing for pathogenic variants (PVs) in BC CSGs has expanded from *BRCA1/BRCA2/PALB2* to incorporate moderate penetrance genes, including *ATM/CHEK2/RAD51C/RAD51D*.^5,6,7^ UK general population women have a lifetime BC risk of 11% up to 80 years of age.^8^ The National Institute for Health and Care Excellence (NICE) familial BC guideline categorizes lifetime BC risk (20 to 80 years of age) of less than 17%, 17% to 30%, and 30% or higher as near population risk, moderate risk, and high risk, respectively.^9^

The identified at-risk women face decision-making regarding BC risk management. BC screening, medical prevention, and risk-reducing surgery are potential management options.^9,10^ The NICE familial BC guideline recommends management strategies based on defined BC risk categories.^9^ Women with a moderate (17%-30%) or high (≥30%) lifetime BC risk are eligible for annual mammography screening.^9^ Women with very high risk (having a 10-year BC risk of 8% at 30 years of age or a BC risk of 12% at 40 years of age) are further eligible for annual magnetic resonance imaging (MRI) screening.^11^ Medical prevention with tamoxifen or anastrozole reduces premenopausal or postmenopausal BC risk, respectively, and is recommended for both women at moderate and high risk levels.^12,13^ Risk-reducing mastectomy (RRM) reduces BC risk by approximately 90%, and guidelines recommend this for women with 30% or higher lifetime BC risk, but is currently clinically only offered to *BRCA1/BRCA2/PALB2* PV carriers in the UK.^9^

RRM is being increasingly undertaken,^14,15,16^ and is cost-effective compared with BC screening and medical prevention among *BRCA1*/*BRCA2*/*PALB2* PV carriers at varying surgery ages.^17,18^ However, its cost-effectiveness at the 30% lifetime BC risk level is unassessed, and the scientific rationale for this BC risk threshold remains unclear. Importantly, the precise lifetime BC risk thresholds at which RRM becomes cost-effective remain undetermined.^18^ Newer moderate penetrance genes with established BC risks (eg, *CHEK2*/*ATM/RAD51C*/*RAD51D*)^19,20^ are now being included in genetic testing panels. This becomes particularly relevant, as although the lifetime BC risk in these moderate penetrance genes themselves is below the risk level for offering RRM, this can be combined with FH or a PRS, which together can lead to an absolute BC risk (for example, 21% BC risk in *RAD51C* can reach 35% to 40% with a first-degree relative with BC and suitable PRS) that lies above a potential newly established risk threshold for RRM in a significant proportion of women.^1,2,21^ Additionally, validated models like Tyrer-Cuzick^4^ and CanRisk^2,3^ can combine epidemiological/reproductive factors, PRS, and mammographic density to provide absolute BC risk estimates to stratify a population by BC risk, with some people falling above a potential BC risk threshold for RRM. We aim to define the lifetime BC risk thresholds for RRM to be cost-effective compared with nonsurgical alternatives for BC prevention.

## Methods

This economic evaluation followed the Consolidated Health Economic Evaluation Reporting Standards (CHEERS) reporting guideline and the NICE health technology evaluations manual,^22^ receiving ethics approval from the London School of Hygiene & Tropical Medicine Ethics Committee. The study was conducted between September 2022 and September 2024.

### Model Overview

**Figure 1.**
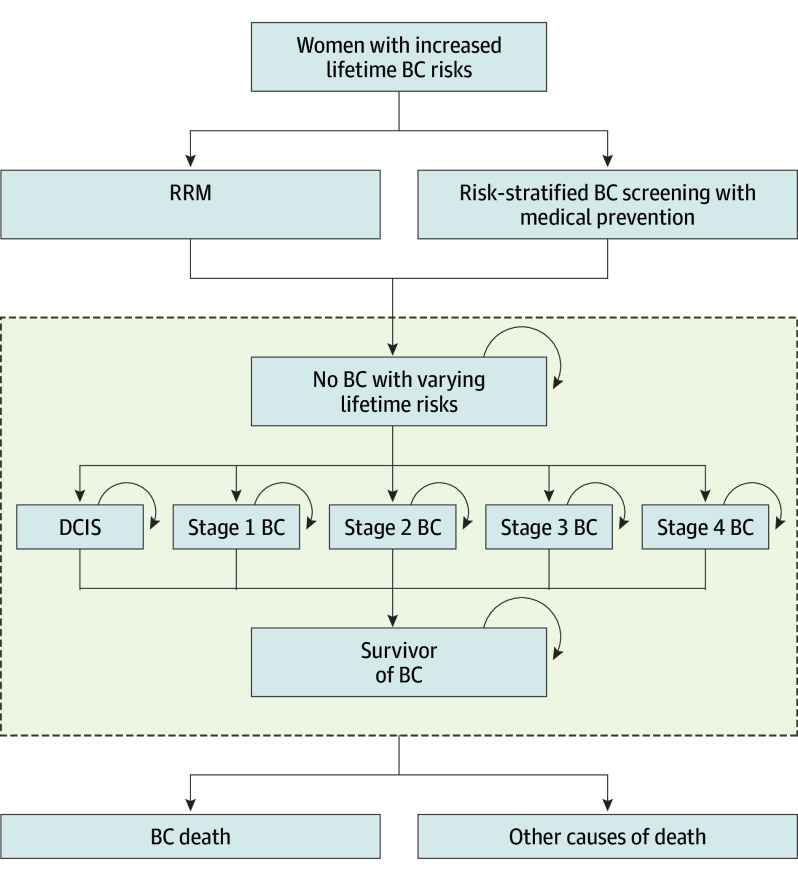
Model Overview The upper part of the diagram shows the decision tree pathway for choosing risk-reducing mastectomy (RRM) or breast cancer (BC) screening. The lower part of the diagram is a schematic illustration of the health states and key transitions for the Markov model. DCIS indicates ductal carcinoma in situ.

A decision analytic Markov model (Figure 1) evaluated the costs and health effects of RRM compared with BC screening for women aged 30 to 60 years at varying lifetime BC risks using TreeAge Pro 2021 (TreeAge Software). The target population was healthy women at increased lifetime BC risks but not *BRCA1/BRCA2/PALB2* PV carriers. They began in a healthy state and progressed through health states of BC stages (ductal carcinoma in situ, stage 1 BC, stage 2 BC, stage 3 BC, and stage 4 BC), BC survivor, cancer-specific death, or all-cause death. BC stage distribution at diagnosis would capture the effects of transitions between different stages up to the point of diagnosis, and our model then tracked the local and distant recurrence and survival over years since diagnosis for each BC stage.^23^ Women diagnosed with BC may die from BC or other causes.

### BC Risk and Age-Specific Incidence

The lifetime BC risk for general population was calculated using the lifetime risk calculator from Cancer Research UK (CRUK),^8^ using data on age-specific BC incidence (2016-2018)^24^ and mortality (2017-2019)^25^ from CRUK, as with female all-cause mortality from Office for National Statistics (2018-2020).^26^ The lifetime BC risk is calculated from age 20 to 80 years for consistency with the NICE definition,^9^ and thus the UK general population women have a lifetime risk of 10.79%. The lifetime average hazard ratio for BC among women with increased BC risk compared to the general population was calculated using the following formula (eTable 1 in Supplement 1)^27^:

Hazard ratio = ln(1 − assumed lifetime BC − risk) ÷ ln(1 − general population lifetime BC − risk)

In this calculation, the “assumed lifetime BC risk” was initially set at a range of different levels: initially 17%, 20%, 25%, 30%, 35%, 40%, 45%, and 50%. These reflect a range of increased BC risk levels that lie between the lower-end threshold of moderate BC risk level established by NICE (lifetime BC risk of ≥17%) and the upper-end level of BC risk associated with *PALB2* carriers (53% BC risk). The general population lifetime BC risk is 10.79% from 20 to 80 years of age for women in the UK. Using these parameters in the formula provided enables hazard ratio calculation. For example, for a 25% lifetime BC risk, hazard ratio = ln(1 − 0.25)/ln(1 − 0.1079) = 2.52, and hazard ratio for 50% lifetime BC risk equals 6.07. The hazard ratio increases with an increase in the assumed lifetime BC risk level. For a given lifetime BC risk level, the hazard ratio was assumed to be constant across the lifetime, given the lack of age-specific estimates for women at increased BC risk.^28,29^ The age-specific incidence of women at increased BC risk was calculated by multiplying the age-specific incidence of the general population by the hazard ratio.^24,28,29^

### Interventions

To estimate the absolute differences across strategies, a 100% uptake of RRM and BC screening was assumed. Women undergo RRM at a plausible age from 30 to 60 years of age. Risk-stratified screening was based on NICE familial BC guideline^9^ and National Health Service (NHS) breast screening program guidance^11^: women with 17% to 32% lifetime BC risk receive annual mammography from 40 to 49 years of age, and thereafter routine triennial mammography until 69 years of age; women with 32% to 45% lifetime BC risk receive annual mammography from 40 to 59 years of age, and thereafter triennial mammography until 69 years of age; women with 45% or higher lifetime BC risk receive annual MRI from 30 to 49 years of age and annual mammography from 50 to 69 years of age. These lifetime BC risk thresholds were converted from the required 10-year risk for mammography and MRI screening.^9,11^ Medical prevention was incorporated for women undergoing screening, and included tamoxifen (premenopausal) or anastrozole (postmenopausal), with an uptake of 16.3%.^30^ Women with 30% or higher lifetime BC risk commence medical prevention at 30 years of age, and those with 17% to 30% lifetime BC risk begin at 40 years of age for 5 years.

### Probabilities

BC risk reduction estimates were derived from the PROSE study for RRM^31^ and the IBIS-I and IBIS-II trials for tamoxifen/anastrozole.^12,13^ BC stage distribution under risk-stratified screening was derived from the Manchester FH risk and prevention clinic.^32^ BC stage distribution from the general population was applied to women who developed BC outside the screening age ranges (ie, outside 40 to 69 years of age for women with a 17% to 45% lifetime risk, or outside 30 to 69 years of age for women with 45% or higher lifetime risk).^33,34^ False-positive recall or biopsy rates for mammography or MRI were taken from a study with 20% to 25% or higher lifetime BC risk.^35^ The proportions of estrogen receptor (ER)–positive, human epidermal growth factor receptor–2 (ERBB2)–positive, and lymph node–positive cancers were taken from the risk-stratified screening Manchester study^32^ and from the general population for women developing BC outside the screening age range.^36^ Recurrence for each BC stage was incorporated.^32,33,36,37,38,39,40^ For detailed probabilities, see eTable 2 and eMethods 1 in Supplement 1.

### Costs

This analysis was conducted from the payer perspective (UK NHS).^22^ The Hospital & Community Health Services index or NHS Cost Inflation Index converted costs to 2021 GBP.^41^ The costs of RRM, MRI/mammography, false positive recall/biopsy, tamoxifen/anastrozole, BC treatments for each stage (first and subsequent years and terminal care) were derived from National Cost Collection for the NHS^42^ and literature (eTable 2 and eMethods 2 in Supplement 1). BC treatment costs were adjusted for proportions being ER-positive, ERBB2-positive, lymph node–positive, or premenopausal for women with varying lifetime BC risks.^32^ Currency conversions were current as of June 18, 2025.^43^

### Life-Years

A lifetime horizon until age 80 years was adopted to align with the NICE guideline definition of lifetime risk. Annual model cycles were used. Women were long-term survivors if alive without recurrence 20 years after diagnosis, facing the same probability of death as the general population.^26^ Stage-specific 20-year survival was derived from the Manchester study for women undergoing screening^32^ and from the general population for those developing BC outside the screening age range.^44^ Survival of women developing BC after RRM was obtained from a cohort of women at increased BC risk.^32^ For detailed survival estimates, see eTable 2 and eMethods 3 in Supplement 1.

### Quality-Adjusted Life-Years

Health state utility values, which adjusted for changes in survival by alterations in quality of life, were used to calculate quality-adjusted life-years (QALYs).^22^ Disutility for RRM was assigned to year of surgery.^45,46,47^ We assigned a 1-week disutility for screening attendance and a 5-week disutility for false-positive results.^46,48^ Disutility for medical prevention was assigned for the treatment duration.^46,47^ Utility values for BC stages were derived from literature.^49,50,51^ See eTable 2 and eMethods 4 in Supplement 1 for a detailed description of disutilities. All utility values were age adjusted using the multiplicative method,^22,52^ combining age-specific utilities in the healthy state^53^ with utilities in all other health states.

### Statistical Analysis

This analysis was conducted from September 1, 2022, to September 30, 2024. The model was validated through a process of face, technical, and cross validity (eMethods 5 in Supplement 1).^54,55^ Costs and health effects were discounted at 3.5% as per NICE guidance.^22^ The incremental cost-effectiveness ratio (ICER) was calculated as the difference in costs divided by the difference in QALYs. Based on the results for initially assumed lifetime BC risks, we adjusted the risk granularity to determine the thresholds at which the ICER fell below NICE willingness-to-pay (WTP) thresholds of £20 000 (US $27 037) to 30 000 (US $40 555) per QALY for RRM compared with BC screening.^56^ BC cases that could potentially be prevented were estimated among women whose risk exceeded the identified threshold at the population level.

Sensitivity analyses evaluated model uncertainty. Parameters were varied individually to assess their impact on ICER in 1-way sensitivity analysis. Probabilities and utility values were varied by their 95% CI/range or by plus or minus 10%, and costs by plus or minus 30%. All parameters were varied simultaneously in probabilistic sensitivity analysis (PSA), with assigned distribution (costs: γ distribution, probabilities: β distribution, utility scores: logarithmic normal distribution^57,58^) over 10 000 simulations. Cost-effectiveness acceptability curves showed the probability that RRM was cost-effective at varying WTP thresholds.

## Results

In the simulated cohort of 100 000 thirty-year-old women in the UK, undergoing RRM was associated with reduced BC incidence and death, with increased costs, life-years, and QALYs for each lifetime BC risk level modeled (Table 1). The cost-effectiveness of RRM improves with higher assumed lifetime BC risk. For 30-year-old women, undergoing RRM became cost-effective at 34% lifetime BC risk (ICER, £28 861 [US $39 016] per QALY) using £30 000 (US $40 555) per QALY WTP threshold. This increased to 42% lifetime BC risk (ICER, £19 553 [US $26 433] per QALY), using £20 000 (US $27 037) per QALY WTP threshold. The identified lifetime BC risk thresholds for RRM cost-effectiveness among women 35, 40, 45, 50, 55, and 60 years of age were 31%, 29%, 29%, 32%, 36%, and 42%, respectively, using £30 000 (US $40 555) per QALY WTP threshold. Using £20 000 (US $27 037) per QALY WTP threshold increased these BC risk thresholds to 37%, 35%, 34%, 38%, 43%, and 50%, respectively (Table 2). The probabilities for RRM being cost-effective among women 30 to 60 years of age from PSA, along with base case ICERs, are summarized in Table 2.

**Table 1.** Lifetime Costs, Health Effects, and Incremental Cost-Effectiveness Ratios of Risk-Reducing Mastectomy for Women 30 Years of Age

Strategy	BC incidence, %	BC death, %	Costs, £ (US$)^a^	LYGs	QALYs	ICER, £/QALY
**17% Lifetime BC risk**						
BC screening	16.21%	1.60%	2446 (3307)	22.91	19.43	
RRM at 30 y of age	1.63%	0.12%	11 926 (16 122)	22.97	19.51	116 698
**20% Lifetime BC risk**						
BC screening	19.06%	1.89%	2754 (3723)	22.89	19.40	
RRM at 30 y of age	1.94%	0.15%	11 957 (16 164)	22.97	19.51	83 282
**25% Lifetime BC risk**						
BC screening	23.89%	2.38%	3283 (4438)	22.87	19.34	
RRM at 30 y of age	2.50%	0.19%	12 013 (16 240)	22.97	19.50	54 135
**30% Lifetime BC risk**						
BC screening	28.62%	2.98%	3715 (5022)	22.85	19.27	
RRM at 30 y of age	3.09%	0.25%	12 059 (16 302)	22.96	19.50	37 390
**32% Lifetime BC risk**						
BC screening	30.58%	3.19%	4144 (5602)	22.84	19.25	
RRM at 30 y of age	3.35%	0.27%	12 084 (16 336)	22.96	19.49	32 104
**34% Lifetime BC risk**						
BC screening	32.49%	3.39%	4352 (5883)	22.83	19.22	
RRM at 30 y of age	3.60%	0.29%	12 108 (16 368)	22.96	19.49	28 861
**36% Lifetime BC risk**						
BC screening	34.42%	3.60%	4565 (6171)	22.82	19.20	
RRM at 30 y of age	3.86%	0.31%	12 133 (16 402)	22.96	19.49	26 044
**38% Lifetime BC risk**						
BC screening	36.35%	3.81%	4780 (6462)	22.81	19.17	
RRM at 30 y of age	4.13%	0.33%	12 159 (16 437)	22.96	19.49	23 580
**40% Lifetime BC risk**						
BC screening	38.22%	4.02%	4991 (6747)	22.80	19.15	
RRM at 30 y of age	4.40%	0.35%	12 185 (16 472)	22.96	19.48	21 481
**42% Lifetime BC risk**						
BC screening	40.17%	4.23%	5212 (7046)	22.79	19.12	
RRM at 30 y of age	4.69%	0.37%	12 213 (16 510)	22.96	19.48	19 553
**44% Lifetime BC risk**						
BC screening	42.11%	4.45%	5436 (7349)	22.77	19.10	
RRM at 30 y of age	4.98%	0.40%	12 242 (16 549)	22.96	19.48	17 839
**45% Lifetime BC risk**						
BC screening	43.03%	4.19%	9296 (12 567)	22.82	19.11	
RRM at 30 y of age	5.13%	0.41%	12 256 (16 568)	22.96	19.48	7963
**50% Lifetime BC risk**						
BC screening	47.88%	4.68%	9835 (13 295)	22.79	19.04	
RRM at 30 y of age	5.92%	0.47%	12 333 (16 672)	22.95	19.47	5853

Abbreviations: BC, breast cancer; ICER, incremental cost-effectiveness ratio; LYG, life-year gained; QALY, quality-adjusted life year; RRM, risk-reducing mastectomy.

^a^ Currency conversion current as of June 18, 2025.

**Table 2.** Cost-Effectiveness of Risk-Reducing Mastectomy Compared With Breast Cancer Screening for Women 30 to 60 Years of Age

Lifetime BC risk	Base case ICER of RRM vs BC screening, £/QALY; probability of being cost-effective^a^						
	Women 30 y of age	Women 35 y of age	Women 40 y of age	Women 45 y of age	Women 50 y of age	Women 55 y of age	Women 60 y of age
17%	116 698; 0.47%	87 310; 1.38%	75 335; 2.80%	73 623; 2.37%	95 069; 0.86%	130 458; 0.22%	221 305; 0.04%
20%	83 282; 1.44%	64 485; 4.29%	56 430; 8.40%	55 227; 7.87%	69 843; 3.09%	93 430; 0.76%	146 783; 0.11%
25%	54 135; 8.60%	43 018; 19.09%	37 981; 30.00%	37 128; 30.36%	46 102; 14.84%	60 378; 5.32%	89 235; 1.08%
28%	43 875; 17.78%	35 086; 36.31%	30 993; 48.13%	30 234; 49.70%	37 325; 30.14%	48 570; 12.59%	70 407; 3.12%
29%	41 196; 21.78%	32 981; 41.54%	29 123; 53.78%^b^	28 385; 57.18%^b^	34 995; 36.61%	45 471; 16.58%	65 604; 3.72%
30%	37 390; 29.09%	30 726; 48.29%	27 570; 60.01%^b^	26 905; 61.77%^b^	33 092; 40.66%	42 938; 19.10%	61 645; 4.89%
31%	35 236; 34.31%	28 937; 54.80%^b^	25 923; 65.48%^b^	25 271; 68.10%^b^	31 047; 48.28%	40 235; 23.69%	57 532; 6.87%
32%	32 104; 43.90%	26 129; 64.97%^b^	23 093; 74.22%^b^	22 217; 77.66%^b^	26 845; 62.18%^b^	35 808; 35.28%	53 084; 9.85%
33%	30 408; 48.77%	24 719; 69.31%^b^	21 803; 78.21%^b^	20 948; 81.48%^b^	25 294; 67.73%^b^	33 721; 40.34%	49 841; 12.29%
34%	28 861; 55.04%^b^	23 429; 74.48%^b^	20 622; 81.79%^b^	19 786; 85.29%^c^	23 876; 71.92%^b^	31 820; 45.28%	46 912; 15.00%
35%	27 443; 59.96%^b^	22 245; 79.08%^b^	19 536; 85.29%^c^	18 716; 88.09%^c^	22 575; 77.12%^b^	30 082; 50.96%	44 253; 17.91%
36%	26 044; 65.27%^b^	21 073; 82.06%^b^	18 461; 88.20%^c^	17 656; 90.30%^c^	21 289; 80.05%^b^	28 368; 56.84%^b^	41 651; 22.96%
37%	24 760; 69.38%^b^	19 997; 84.98%^c^	17 472; 90.61%^c^	16 681; 91.92%^c^	20 108; 83.66%^b^	26 798; 62.39%^b^	39 284; 27.29%
38%	23 580; 74.16%^b^	19 005; 87.74%^c^	16 559; 92.29%^c^	15 781; 93.80%^c^	19 020; 86.88%^c^	25 356; 67.50%^b^	37 122; 31.03%
40%	21 481; 81.29%^b^	17 236; 91.60%^c^	14 932; 94.55%^c^	14 174; 95.67%^c^	17 081; 90.93%^c^	22 796; 76.49%^b^	33 317; 41.42%
42%	19 553; 87.36%^c^	15 607; 94.67%^c^	13 431; 95.96%^c^	12 691; 97.19%^c^	15 297; 94.60%^c^	20 449; 83.79%^b^	29 862; 51.40%^b^
43%	18 692; 89.36%^c^	14 878; 95.53%^c^	12 758; 97.04%^c^	12 026; 97.65%^c^	14 499; 95.10%^c^	19 403; 85.82%^c^	28 332; 56.28%^b^
44%	17 839; 91.14%^c^	14 154; 96.38%^c^	12 091; 97.69%^c^	11 366; 97.95%^c^	13 707; 95.88%^c^	18 367; 88.20%^c^	26 824; 62.10%^b^
45%	7963; 98.57%^c^	7244; 99.05%^c^	7248; 99.24%^c^	8269; 99.08%^c^	12 366; 97.40%^c^	16 402; 91.69%^c^	23 579; 72.00%^b^
50%	5853; 99.35%^c^	5220; 99.56%^c^	5179; 99.65%^c^	5990; 99.75%^c^	9332; 99.02%^c^	12 493; 96.53%^c^	18 060; 88.20%^c^

Abbreviations: BC, breast cancer; ICER, incremental cost-effectiveness ratio; PSA, probabilistic sensitivity analysis; QALY, quality-adjusted life year; RRM, risk-reducing mastectomy.

^a^ Using £30 000 per QALY threshold in the PSA.

^b^ ICER < £30 000/QALY.

^c^ ICER < £20 000/QALY.

**Figure 2.**
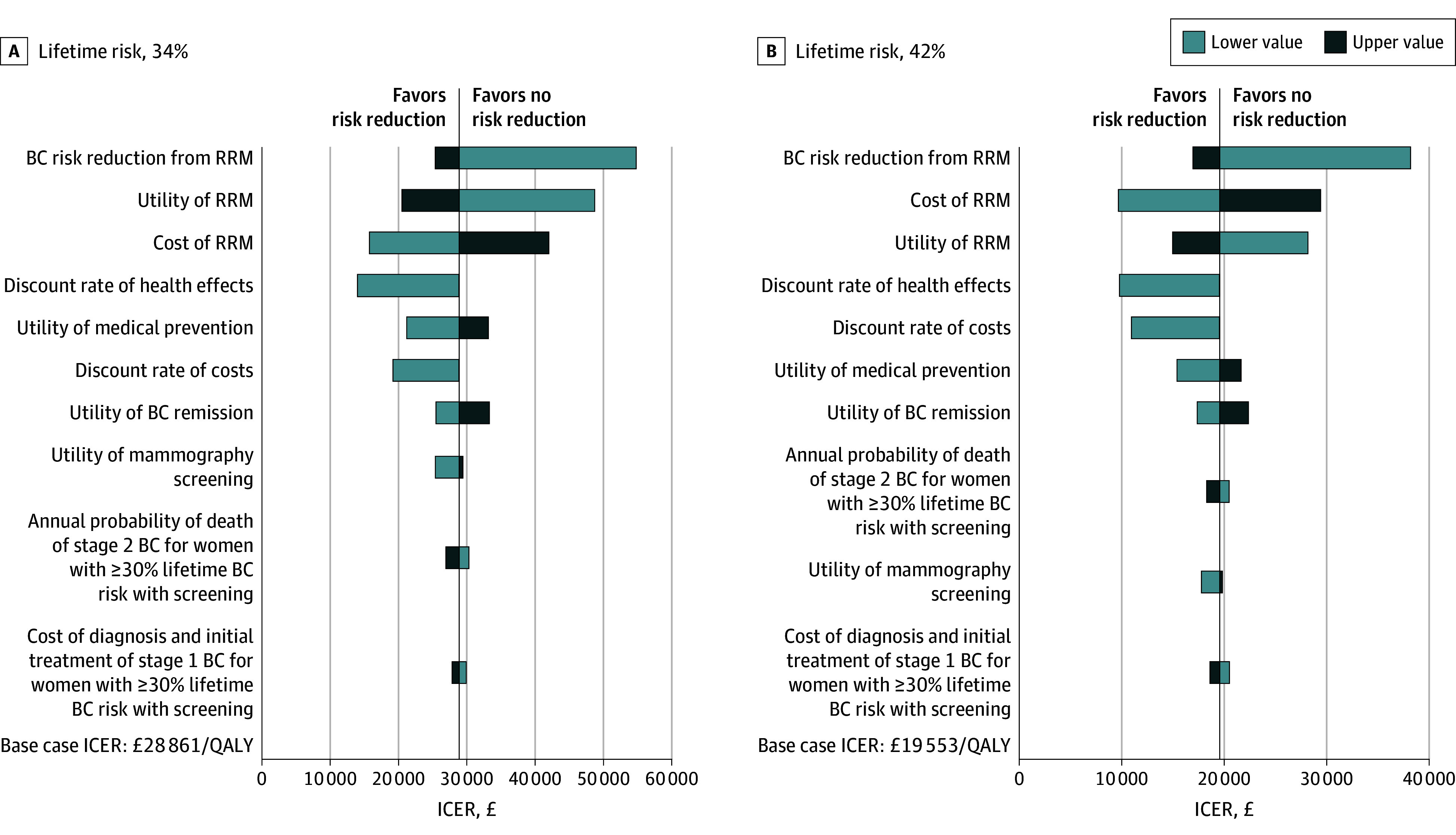
Tornado Diagrams of 1-Way Sensitivity Analyses A, Risk-reducing mastectomy (RRM) vs breast cancer (BC) screening for women aged 30 years with a 34% lifetime risk. B, RRM vs BC screening for women aged 30 years with a 42% lifetime risk. ICER indicates incremental cost-effectiveness ratio; QALY, quality-adjusted life years.

**Figure 3.**
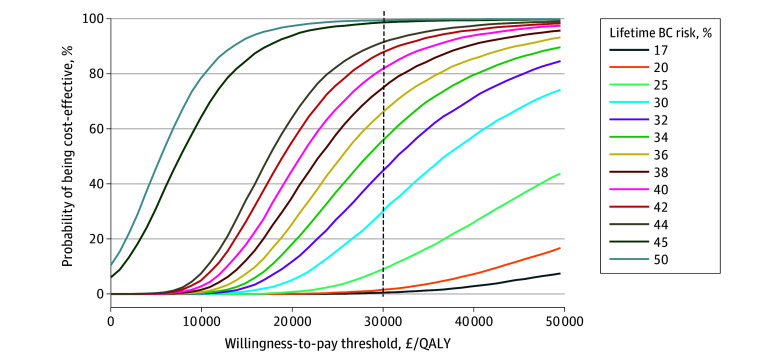
Cost-Effectiveness Acceptability Curves for Risk-Reducing Mastectomy in Women Aged 30 Years BC indicates breast cancer; QALY, quality-adjusted life-years.

The 1-way sensitivity analyses (Figure 2) showed that risk reduction effect, disutility, and RRM costs had the largest influence on base case results. The discount rate for health effects exerted a larger influence than the discount rate for costs. The influence of parameters on the cost-effectiveness of RRM decreases as the modeled lifetime BC risk increases. The probability of RRM being cost-effective compared with BC screening increased with lifetime BC risk in the PSA (Table 2, Figure 3). For 30-year-old women, 59.96%, 81.29%, 98.57%, and 99.35% of simulations were cost-effective for women at 35%, 40%, 45%, and 50% lifetime BC risks undergoing RRM using £30 000 (US $40 555) per QALY WTP threshold.

Overall, undergoing RRM was deemed cost-effective for women 30 to 55 years of age with a lifetime BC risk of 35% or higher, with more than 50% of PSA simulations being cost-effective (Table 2). Approximately 3% of women in the general population in the UK have a lifetime BC risk of 35% or higher.^59^ This includes *BRCA1/BRCA2* PV carriers and other women with increased risk. The general population prevalence of women carrying *BRCA1/BRCA2* is approximately 0.5%,^60^ and hence, prevalence of other women with increased risk is 2.5%. The weighted average lifetime BC risk is approximately 60% for *BRCA1/BRCA2* PV carriers^61^ and approximately 41.8% for other women with increased risk.^62^ Given the lifetime risk of approximately 11% in the general population, the proportion of BC cases from women with a 35% or higher lifetime BC risk is calculated with the following equation: (60% × 0.5% + 41.8% × 2.5%)/11% = 12.23%. Offering RRM for women with lifetime BC risks of 35% or higher could potentially prevent 6538 (95% CI, 4454-7041) or approximately 11% (95% CI, 8%-12%) of the 58 756 BC cases occurring annually in women in the UK.^31,63^

## Discussion

This economic evaluation defines the lifetime BC risk thresholds for RRM cost-effectiveness compared with screening/medical prevention for BC prevention. While the identified lifetime BC risk thresholds varied by age, undergoing RRM appears cost-effective for women aged 30 to 55 years with a lifetime BC risk of 35% or higher. Offering RRM to all women in the UK at 35% or higher lifetime BC risk can potentially prevent approximately 6500 BC cases annually. Validated BC risk models can identify individuals above this risk level, and population stratification for BC risk is being evaluated in clinical trials.^64,65^ Our results support personalized BC risk prediction for both moderate penetrance BC genes and potentially population stratification strategies. This will enable counseling for RRM and management recommendations based on women’s age and individualized BC risk. This approach could expand access to RRM beyond the traditional *BRCA1/BRCA2/PALB2* PV carriers for women at 35% or higher lifetime BC risk. Nonsurgical alternatives, including screening and medical prevention, would remain for those at more moderate (17% to 34.9%) lifetime BC risks.

A 20-year RRM uptake of 48% among *BRCA1/BRCA2* PV carriers and up to 9% among non-CSG carriers with 30% or higher lifetime BC risk was reported by the Manchester high-risk prevention clinic.^14^ However, previously, RRM access for non-CSG carriers has been rare, patchy, and lacked clinical consensus. Our suggested RRM BC risk threshold is higher than the current 30% NICE recommendation. NICE/clinical guidelines should consider changes to reflect this new RRM BC risk threshold. An increase in RRM uptake resulting from increased access, awareness, and availability may have considerable resource implications, especially with complex reconstruction procedures.^14,66^ This needs to be addressed along with expansion in the clinician service provision. We previously identified lifetime ovarian cancer (OC) risk thresholds (≥4%-5% lifetime OC risk) for risk-reducing salpingo-oophorectomy, which have now been incorporated into guidelines, broadening access to surgical prevention in the UK (and internationally).^67,68,69,70,71^

Large-scale NHS programs like PROCAS^64^ and BC-PREDICT^72^ have demonstrated the feasibility and acceptability of risk-stratified BC screening, which predicts individualized BC risks using validated risk models like Tyrer-Cuzick or CanRisk. International randomized clinical trials (RCTs) are currently evaluating this approach.^65,73^ A proportion of these women will fall above the 35% lifetime risk threshold for RRM.

Women with a strong FH of BC are offered panel genetic testing, which includes moderate penetrance genes like *ATM/CHEK2/RAD51C/RAD51D*.^74^ This is now being advocated for unselected (all) women at BC diagnosis.^5,74^ Panel testing for patients with OC also includes moderate penetrance BC CSGs (eg, *RAD51C/RAD51D*).^75,76^ Many more unaffected moderate BC CSG PV carriers will be identified through future cascade testing. The lifetime BC risk for women in the PRS 90th percentile reached 40.9% for *ATM* carriers and 46.6% for *CHEK2* carriers with FH, respectively.^1^ A first-degree relative with BC in a *CHEK2* carrier itself increases lifetime BC risk to 33%.^2^ *RAD51C* and *RAD51D* PV carriers with 2 first-degree relatives with BC may have lifetime BC risks of 46% and 44%, respectively, while BC risks were 34% and 32% with 1 first-degree relative with BC, respectively.^77^ Addition of PRS and/or other risk factors further improves the precision of BC risk estimation.^1,2,78^ Our identified (≥35%) lifetime BC risk thresholds make RRM a potential option for moderate penetrance gene carriers with confirmed risk modifiers such as FH or PRS,^79,80^ thereby potentially avoiding future health system cancer treatment costs. For women with a lifetime BC risk ≤35%, recommendations of BC screening and/or medical prevention facilitate risk management and avoid adverse events/additional surgical costs.

### Strengths and Limitations

Our study has several strengths. For the first time, to our knowledge, this study identified the lifetime BC risk thresholds for RRM cost-effectiveness. An RCT comparing RRM with BC screening is unfeasible given ethical issues and lack of patient acceptability. The modeling approach adopted can simulate long-term outcomes of these strategies. We explored lifetime BC risk thresholds from 17% to 50% and varying surgery ages from 30 to 60 years. We used risk-stratified BC screening and medical prevention as the comparator instead of “no intervention” to yield more conservative BC risk threshold estimates. UK-specific data on risk-stratified BC screening were used.^32^

Our study has several limitations. We assumed constant average hazard ratio for women with increased BC risk due to the lack of age-specific estimates, which need updating when data become available. Lack of prospective data on the level of risk reduction from RRM for non-*BRCA* carriers at increased BC risk leads us to use estimates from *BRCA1/BRCA2* PV carriers.^31^ To address uncertainty, we conducted sensitivity analyses for a wide range of this parameter (62% to 98%). The evidence regarding the effect of RRM on BC mortality is not conclusive, with both no improvement^81,82^ or reduced mortality for *BRCA1* PV carriers^83^ reported. Our BC risk threshold estimates were conservative, as we assumed no mortality benefit from RRM. Although UK data on risk-stratified screening were used,^32^ parameters for stage distribution, pathology, and survival can only be categorized for women with 17% to 30% and 30% or higher lifetime BC risks due to limited sample size (N = 394), which did not exactly match our modeled screening strategy. Our sensitivity analyses showed minimal impacts from these parameters (Figure 2). Furthermore, potential harms for each intervention may not be fully captured despite the disutilities assigned. RRM utility values derived from EQ-5D data (recommended by NICE) are lacking; therefore, we used estimates from time trade-off surveys.^46,47^ Future research measuring long-term RRM utility values from EQ-5D will improve the precision of risk threshold estimates.

Screening recommendations and model parameters may vary across health systems, potentially limiting direct extrapolation of findings. Women in the US with a lifetime BC risk of 20% or higher undergo more intensive screening^9,11^ (annual mammography and MRI from 30 years)^84^; and health system costs of screening/RRM/cancer treatment/medical procedures are higher compared with the UK.^5^ However, this is partly offset by higher WTP thresholds ($100 000/QALY). Nonetheless, we have in other contexts found similar cost-effectiveness for UK and US health systems.^5,85,86,87^ Similar methodological frameworks can identify lifetime BC risk thresholds for RRM in other health systems.

Existing BC risk models (eg, Tyrer-Cuzick/CanRisk) may,^88^ overestimate lifetime BC risk among women with high-risk breast lesions (atypical hyperplasia/lobular carcinoma in situ)^89^; be less accurate in racial/ethnic (vs White) populations; and be better validated for 10-year (vs lifetime) BC risks. These limitations should be considered when assessing BC risk and RRM eligibility. We modeled lifetime BC risk to capture long-term lifetime costs and consequences of RRM and align with guidelines, which base recommendations on lifetime risk.^9,90^

Women at increased BC risk should be given detailed information and counseling on the risks and benefits of RRM, along with alternative options for BC screening and medical prevention.^91,92^ The negative impacts on body image and sexual function, the possibility of complications (approximately 20% with reconstruction), and unanticipated additional surgical procedures following RRM should be factored into counseling. Decision aids/tools to facilitate understanding of risk and informed consent are needed.^45,91,92,93^ The varying lifetime BC risk thresholds identified for different ages reflect the trade-off between the costs/disutility of RRM and future cancer risks, aiding women in making personalized decisions regarding optimal timing of RRM. Our identified lifetime BC risk thresholds for RRM contribute to the evidence base for personalized management of women at moderate risk of BC/OC. Future studies on acceptability, uptake, and impact of RRM are needed among (non–*BRCA1/BRCA2/PALB2* carriers) women with 35% or higher lifetime BC risk. Referral and care pathways incorporating all stakeholders, including general practitioners, genetics clinicians/counsellors, breast specialists, psychologists, and care commissioners, need to be expanded/developed.

## Conclusions

In this economic evaluation, the identified lifetime BC risk thresholds for RRM cost-effectiveness varied by age. Undergoing RRM appears cost-effective for women 30 to 55 years of age with a lifetime BC risk of 35% or higher. The results could have significant clinical implications to expand access to RRM beyond *BRCA1/BRCA2/PALB2* PV carriers, and could potentially prevent 6500 BC cases annually. Future studies evaluating the acceptability, uptake, and long-term impact of RRM among these women are needed.

## Notes

Supplement 1. eTable 1. Hazard ratio for breast cancer among women with increased lifetime risks compared to general population eTable 2. Model parameters eMethods 1. Detailed probabilities calculation eMethods 2. Detailed costs calculation eMethods 3. Survival of breast cancer and impact of interventions eMethods 4. Detailed utility value calculation eMethods 5. Model validation eReferences

Supplement 2. Data sharing statement
