# Supplementary material for: Defining Lifetime Risk Thresholds for Breast Cancer Surgical Prevention
Source: JAMA Oncol. 2025 Jul 24;11(9):1072–82. doi: 10.1001/jamaoncol.2025.2203 (PMC12290908; doi:10.1001/jamaoncol.2025.2203)
Supplement: Supplement 1. — eTable 1. Hazard ratio for breast cancer among women with increased lifetime risks compared to general population eTable 2. Model parameters eMethods 1. Detailed probabilities calculation eMethods 2. Detailed costs calculation eMethods 3. Survival of breast cancer and impact of interventions eMethods 4. Detailed utility value calculation eMethods 5. Model validation eReferences [file jamaoncol-e252203-s001.pdf]

Supplemental Online Content

Wei X, Mansour L, Oxley S, et al. Defining lifetime risk thresholds for breast cancer surgical prevention. *JAMA Oncol.* Published online July 24, 2025.
doi:10.1001/jamaoncol.2025.2203

eTable 1. Hazard ratio for breast cancer among women with increased lifetime risks compared to general population

eTable 2. Model parameters

eMethods 1. Detailed probabilities calculation

eMethods 2. Detailed costs calculation

eMethods 3. Survival of breast cancer and impact of interventions

eMethods 4. Detailed utility value calculation

eMethods 5. Model validation

eReferences

This supplemental material has been provided by the authors to give readers additional information about their work.

© 2025 Wei X et al. *JAMA Oncology*.

eTable 1. Hazard ratio for breast cancer among women with increased lifetime risks compared to general population

**Lifetime BC-risk (age 20 to 80 years)**	**Average hazard ratio for BC compared to general population**
10.79% (UK general population women)	Reference
17%	1.63
20%	1.95
25%	2.52
26%	2.64
27%	2.76
28%	2.88
29%	3.00
30%	3.12
31%	3.25
32%	3.38
33%	3.51
34%	3.64
35%	3.77
36%	3.91
37%	4.05
38%	4.19
39%	4.33
40%	4.47
41%	4.62
42%	4.77
43%	4.92
44%	5.08
45%	5.23
46%	5.40
47%	5.56
48%	5.73
49%	5.90
50%	6.07

Abbreviation: BC, breast cancer

© 2025 Wei X et al. *JAMA Oncology*.

eTable 2. Model parameters

Parameters	Base case	95%CI or ranges	Distribution	Source
**Probabilities**				
Effectiveness of interventions				
BC risk-reduction from RRM	0.91	0.62–0.98	Beta	Rebbeck et al[1]
False positive recall rate of mammography screening	0.0471	0.0397–0.0557	Beta	Vreemann et al[2]
False positive recall rate of MRI screening	0.0661	0.0580–0.0753	Beta	
False positive biopsy rate of mammography screening	0.0250	0.0199–0.0312	Beta	
False positive biopsy rate of MRI screening	0.0492	0.0424–0.0570	Beta	
Uptake of medical prevention	0.163	0.136–0.190	NA	Smith et al[3]
HR for ER-positive BC from tamoxifen	0.66	0.54–0.81	Beta	Cuzick et al[4]
HR for ER-positive BC from anastrozole	0.46	0.33–0.65	Beta	Cuzick et al[5]
Cancer characteristics				
Stage distribution for women with 17%–30% lifetime risk under screening				Evans et al[6]; Brown et al[7]
DCIS	0.2034	NA	NA	
Stage 1 BC	0.4576	NA	NA	
Stage 2 BC	0.2899	NA	NA	
Stage 3 BC	0.0188	NA	NA	
Stage 4 BC	0.0303	NA	NA	
Stage distribution for women with ≥30% lifetime risk under screening				
DCIS	0.2000	NA	NA	
Stage 1 BC	0.5436	NA	NA	
Stage 2 BC	0.2414	NA	NA	
Stage 3 BC	0.0055	NA	NA	
Stage 4 BC	0.0096	NA	NA	

© 2025 Wei X et al. *JAMA Oncology*.

Parameters	Base case	95%CI or ranges	Distribution	Source
Stage distribution for women without screening (outside screening age range)				NICE[8]; Cancer Research UK[9]
DCIS	0.1000	NA	NA	
Stage 1 BC	0.3960	NA	NA	
Stage 2 BC	0.3735	NA	NA	
Stage 3 BC	0.0819	NA	NA	
Stage 4 BC	0.0486	NA	NA	
Annual recurrence probability of BC				
DCIS	0.0124	NA	NA	Evans et al[6]; Wapnir et al[10]; Anderson et al[11]; Gennari et al[12]; Mavaddat et al[13]; NICE[8,14]
Stage 1–3 BC for women with 17%–30% lifetime risk under screening	0.0234	NA	NA	
Stage 1–3 BC for women with ≥30% lifetime risk under screening	0.0232	NA	NA	
Stage 1–3 BC for women without screening	0.0236	NA	NA	
Stage 4 BC	0.0326	NA	NA	
BC survival				
20-year survival for DCIS	0.9670	0.9640–0.9700	Beta	Narod et al[15]
5-year survival for general population				
Stage 1 BC	0.982	NA	NA	Cancer Research UK[16]
Stage 2 BC	0.895	NA	NA	
Stage 3 BC	0.722	NA	NA	
Stage 4 BC	0.266	NA	NA	
20-year survival for general population (estimated, see eMethods 3)				
Stage 1 BC	0.9299	0.8369–0.9999	Beta	Cancer Research UK[16]
Stage 2 BC	0.6416	0.5775–0.7058	Beta	
Stage 3 BC	0.2717	0.2446–0.2989	Beta	
Stage 4 BC	0.0050	0.0045–0.0055	Beta	

© 2025 Wei X et al. *JAMA Oncology*.

Parameters	Base case	95%CI or ranges	Distribution	Source
10-year survival for women with ≥17% lifetime risk under screening	0.9076	0.8466 –0.9452	NA	Evans et al[6]
20-year survival for women with 17%-30% lifetime risk under screening (estimated, see eMethods 3)				
Stage 1 BC	0.9682	0.9460–0.9814	Beta	Evans et al[6]; Cancer Research UK[16]; Brown et al[7]
Stage 2 BC	0.8208	0.7124–0.8916	Beta	
Stage 3 BC	0.5600	0.3694–0.7139	Beta	
Stage 4 BC	0.0947	0.0175–0.2541	Beta	
20-year survival for women with ≥30% lifetime risk under screening (estimated, see eMethods 3)				
Stage 1 BC	0.9465	0.9098–0.9685	Beta	Evans et al[6]; Cancer Research UK[16]; Brown et al[7]
Stage 2 BC	0.7146	0.5615–0.8225	Beta	
Stage 3 BC	0.3728	0.1836–0.5635	Beta	
Stage 4 BC	0.0181	0.0010–0.0971	Beta	
All-cause mortality	National female life tables	NA	NA	Office for National Statistics[17]
**Costs (2021 GBP)**				
RRM (with reconstruction and complication)	11,768	8,238–15,298	Gamma	NHS reference costs[18]; Neuburger et al[19]; Del Corral et al[20]
Annual tamoxifen cost (premenopausal)	36	25–47	Gamma	BNF[21]
Annual anastrozole cost (postmenopausal)	14	10-18	Gamma	BNF[21]
BC screening				
Clinical breast examination	250	175–325	Gamma	NHS reference costs[18]; NICE[22]
Mammography	60	42–79	Gamma	
MRI	254	178–331	Gamma	
Ultrasound guided core needle biopsy of lesion of breast	551	386–716	Gamma	
Diagnosis and initial treatment cost of BC				

© 2025 Wei X et al. *JAMA Oncology*.

Parameters	Base case	95%CI or ranges	Distribution	Source
DCIS				NHS reference costs[18]; NICE[8,22]; Evans et al[6]; Sun et al[23]; Jeevan et al[24]; Miller et al[25]; Del Corral et al[20]; Mavaddat et al[13]
Women with 17%–30% lifetime risk under screening	9,167	6,417–11,917	Gamma	
Women with ≥30% lifetime risk under screening	9,167	6,417–11,917	Gamma	
Women without screening	9,167	6,417–11,917	Gamma	
Stage 1 BC				
Women with 17%–30% lifetime risk under screening	19,774	13,842–25,707	Gamma	
Women with ≥30% lifetime risk under screening	19,621	13,735–25,508	Gamma	
Women without screening	19,959	13,971–25,947	Gamma	
Stage 2 BC				
Women with 17%–30% lifetime risk under screening	21,771	15,240–28,302	Gamma	
Women with ≥30% lifetime risk under screening	21,621	15,135–28,108	Gamma	
Women without screening	21,951	15,366–28,537	Gamma	
Stage 3 BC				
Women with 17%–30% lifetime risk under screening	28,179	19,725–36,633	Gamma	
Women with ≥30% lifetime risk under screening	27,997	19,598–36,396	Gamma	
Women without screening	28,399	19,879–36,919	Gamma	
Stage 4 BC				
Women with 17%–30% lifetime risk under screening	41,466	29,026–53,905	Gamma	
Women with ≥30% lifetime risk under screening	41,466	29,026–53,905	Gamma	
Women without screening	41,466	29,026–53,905	Gamma	
Annual treatment cost of BC 1 to 20 years after diagnosis				
DCIS				NHS reference costs[18]; BNF[21]; NICE[8,22,26-28]; Evans et al[6]; Sun et al[29]; Coleman et al[30]; Mavaddat et al[13]
Women with 17%–30% lifetime risk under screening	402	282–523	Gamma	
Women with ≥30% lifetime risk under screening	399	279–519	Gamma	
Women without screening	403	282–523	Gamma	

© 2025 Wei X et al. *JAMA Oncology*.

Parameters	Base case	95%CI or ranges	Distribution	Source
Stage 1 BC				
Women with 17%–30% lifetime risk under screening	634	444–825	Gamma	
Women with ≥30% lifetime risk under screening	597	418–776	Gamma	
Women without screening	640	448–832	Gamma	
Stage 2 BC				
Women with 17%–30% lifetime risk under screening	634	444–825	Gamma	
Women with ≥30% lifetime risk under screening	597	418–776	Gamma	
Women without screening	640	448–832	Gamma	
Stage 3 BC				
Women with 17%–30% lifetime risk under screening	634	444–825	Gamma	
Women with ≥30% lifetime risk under screening	597	418–776	Gamma	
Women without screening	640	448–832	Gamma	
Stage 4 BC				
Women with 17%–30% lifetime risk under screening	1,014	710–1,319	Gamma	
Women with ≥30% lifetime risk under screening	973	681–1,265	Gamma	
Women without screening	1,018	713–1,324	Gamma	
Terminal care of BC	14,277	1,408–43,648	Gamma	Round et al[31]
**Utility values**				
Population norms (age-adjusted)	0.692–0.913	0.672–0.936	Log-normal	Szende et al[32]
RRM	0.880	0.760–1.000	Log-normal	Grann et al[33,34]
Medical prevention	0.950	0.810–1.000	Log-normal	
Mammography screening	0.970	0.754–1.000	Log-normal	Grann et al[34]
MRI screening	0.960	0.764–1.000	Log-normal	
Disutility from false positive result of BC screening	0.105	0.095–0.116	Log-normal	Geuzinge et al[35]; De Haes et al[36]

© 2025 Wei X et al. *JAMA Oncology*.

**Parameters**	**Base case**	**95%CI or ranges**	**Distribution**	**Source**
BC				
DCIS	0.712	0.640–0.783	Log-normal	Robertson et al[37]
Stage 1 BC	0.597	0.537–0.656	Log-normal	
Stage 2 BC	0.527	0.474–0.580	Log-normal	
Stage 3 BC	0.497	0.447–0.547	Log-normal	
Stage 4 BC	0.394	0.354–0.433	Log-normal	
BC recurrence	0.450	0.215–0.685	Log-normal	Cooper et al[38]
BC remission	0.810	0.771–0.849	Log-normal	
Terminal stage of BC	0.160	0.144–0.176	Log-normal	Peasgood et al[39]

Abbreviation: BC, breast cancer; BNF, British National formulary; CI, confidence interval; DCIS, ductal carcinoma in situ; ER, oestrogen receptor; HR, hazard ratio; NA, not applicable; NHS, National Health Service; NICE, National Institute for Health and Care Excellence; RRM, risk-reducing mastectomy.

© 2025 Wei X et al. *JAMA Oncology*.

eMethods 1. Detailed probabilities calculation

Effectiveness of interventions

Risk-reducing mastectomy (RRM) reduces breast cancer (BC) risk by 91% (95%CI: 62%, 98%) based on the PROSE study for *BRCA1/BRCA2* pathogenetic variant (PV) carriers.[1] This estimate was applied in the model due to the lack of data for other women at increased lifetime BC risks. Invasive estrogen receptor (ER)-positive BC risk was reduced by 34% (hazard ratio 0.66; 95%CI: 0.54, 0.81) and 54% (hazard ratio 0.46; 95%CI: 0.33, 0.65) for tamoxifen or anastrozole, respectively, derived from the IBIS-I[4] and IBIS-II[5] trials.

Based on the study on 14,311 women from the family history risk and prevention clinic in Manchester,[6] the number of women with 17%–30% lifetime BC risk receiving mammography screening diagnosed as stage 1 BC, stage 2 BC, stage 3 BC, and stage 4 BC was 27 (57.45%), 18 (38.30%), 2 (4.26%) and 0 (0%). respectively.[6] There were no stage 4 BC reported due to small number of cases in the study;[6] however at a population level one would expect at least a small number of stage 4 cases. In the general population, around 5% are diagnosed at stage 4 BC.[9] A Wilson 95% confidence interval on the proportion of stage 4 BC would be from 0.1% to 7.4%.[7] We therefore assumed that at a population level, one would expect to observe the mid-point of this interval, that is 3.8% at stage 4 BC. We redistributed 50% each of these from stage 2 and stage 3 BC, giving proportions for stages 1–4 BC as 57.45%, 36.40%, 2.36%, and 3.80% respectively. The proportion of ductal carcinoma in situ (DCIS), stage 1 BC, stage 2 BC, stage 3 BC, and stage 4 BC was 20.34%, 45.76%, 28.99%, 1.88%, and 3.03% for women with 17%-30% lifetime BC risk receiving mammography screening.[6] For women with ≥30% lifetime BC risk receiving mammography with or without MRI screening, 106 (67.95%), 48 (30.77%), 2 (1.28%), and 0 (0%) were diagnosed at stage 1 BC, stage 2 BC, stage 3 BC, and stage 4 BC respectively.[6] Similar assumptions were applied as no stage 4 BC reported. The Wilson 95% confidence interval on

© 2025 Wei X et al. *JAMA Oncology*.

the proportion of stage 4 BC was from 0 to 2.4%.[7] Taking the mid-point of this interval (1.2%) and redistributing half of this figure from stage 2 and 3 BC, we have proportions of 67.95%, 30.17%, 0.68% and 1.20% for stages 1–4 BC respectively. The proportion of DCIS, stage 1 BC, stage 2 BC, stage 3 BC, and stage 4 BC was 20.00%, 54.36 %, 24.14%, 0.55%, 0.96% for women with ≥30% lifetime BC risk receiving mammography with or without MRI screening.[6] BC stage distribution of general population was applied to women developing BC outside the screening age range (DCIS: 10.00%, stage 1 BC: 39.60%, stage 2 BC: 37.35%, stage 3 BC: 8.19%, stage 4 BC: 4.86%).[8,9] False positive results of screening were included, taken from the study on women with lifetime BC risk of ≥20%–25% in the Netherlands.[2] The false positive recall rates using mammography and MRI were 4.71% (95%CI: 3.97%, 5.57%) and 6.61% (95%CI: 5.80, 7.53%), and the false positive biopsy rates were 2.50% (95%CI: 1.99%, 3.12%) and 4.92% (95%CI: 4.24%, 5.70%), respectively.[2]

Pathology characteristics of BC

The proportions of ER-positive, human epidermal growth factor receptor 2 (HER2)-positive, and lymph node positive BC were also derived from the Manchester study for women with increased lifetime BC risk under screening[6] and from general population for women developing BC outside the screening age range.[13] 78.00%, 76.76%, 81.00% BC were ER-positive; 18.18%, 9.52%, and 19.00% BC were HER2-positive; and 26.92%, 19.41%, and 36.00% BC were lymph node positive for women with 17%–30% lifetime BC risk under screening, women with ≥30% lifetime BC risk under screening, and women with no screening, respectively. 49% of BC cases were assumed to be premenopausal.[29]

BC recurrence

Recurrence was modelled for each BC stage since diagnosis. For DCIS, there was a 25% risk of local recurrence over 10 years and half of these recurrences would be invasive cancer.[14] For early and locally advanced BC, the locoregional recurrence rate was 15.9% for node-

© 2025 Wei X et al. *JAMA Oncology*.

positive cancer after 13.3 years follow-up,[10] and 11.0% for node-negative cancer after 16.1 years follow-up.[11] Based on the proportion of being lymph node positive BC,[6,13] the weighted locoregional recurrence rate for early and locally advanced BC was 12.32%, 11.95%, and 12.76% for women with 17%–30% lifetime BC risk under screening, women with ≥30% lifetime BC risk under screening, and women with no screening, respectively. Approximately 35% of the early and locally advanced BC would progress to advanced BC.[8] Advanced BC has a reported recurrence rate of 66.3%, based on a 33.7% relapse-free survival rate over 5 years.[12] The combined recurrence rate for DCIS, stage 1–3 BC, stage 4 BC was 25.00%, 47.32%, and 66.30% for women with 17%–30% lifetime BC risk under screening, 25.00%, 46.95 %, and 66.30% for women with ≥30% lifetime BC risk under screening, and 25.00%, 47.76%, and 66.30% for women with no screening.

© 2025 Wei X et al. *JAMA Oncology*.

eMethods 2. Detailed costs calculation

UK costs data were used wherever possible, and the Hospital & Community Health Services index or NHS Cost Inflation Index were used to convert costs to the year of 2021.[40] Where UK data was unavailable, we used consumer price index (CPI)[41] of original currency and purchasing power parities (PPP)[42] to convert data to 2021GBP.

Costs of interventions

RRM

The costs of RRM and reconstruction were derived from National Cost Collection for the NHS (finial year 2020/2021, the average unit cost of providing defined services to NHS patients in England).[18] The cost of RRM was based on the procedure cost for Healthcare Resource Groups (HRG) code JA21B (Bilateral Major Breast Procedures with CC Score 0), while the cost for RRM and reconstruction was based on the procedure cost for HRG code JA33Z (Bilateral Excision of Breast with Immediate Pedicled Myocutaneous Flap Reconstruction). Around 90.6% reconstruction rate has been reported after RRM in the UK population.[19] For RRM and reconstruction, the minor and major complication rate was reported to be 26.2% and 5.6%, respectively.[20] The complication related costs were added to the costs of RRM (minor complication: £475, major complication: £4,331).[20]

BC screening

The costs of clinical breast examination, mammography, MRI, ultrasound guided core needle biopsy were derived from National Cost Collection for the NHS[18] and NICE familial BC costing report.[22] The additional costs of clinical assessment due to false positive recall and additional costs of biopsy due to false positive biopsy were included for mammography and MRI screening.[2]

Medical prevention

© 2025 Wei X et al. *JAMA Oncology*.

Tamoxifen 20mg daily or anastrozole 1mg daily was given for 5 years for medical prevention, with an uptake rate of 16.3% (obtained from a meta-analysis).[3] The unit cost of tamoxifen and anastrozole was taken from BNF.[21]

Costs of diagnosis, treatment, and terminal care of BC

BC costs were adjusted by the proportions of being ER-positive, HER2-positive, lymph node positive, or premenopausal for women with varying lifetime BC risks.

Diagnosis costs

BC diagnosis was made by the combination of clinical examination, mammography, and biopsy. For patients with suspected advanced cancer, MRI should be offered to assess for bone metastases.[26] The unit cost of clinical examination, mammography, MRI, and ultrasound guided core needle biopsy was derived from National Cost Collection for the NHS[18] and NICE familial BC costing report.[22]

Pre-treatment axilla ultrasound costs

Pretreatment ultrasound evaluation of the axilla should be offered to all patients being investigated for early invasive cancer.[28] Ultrasound guided needle biopsy should be offered if morphologically abnormal lymph nodes are identified (around 33% of women with early invasive BC).[8,28] The commissioning cost of pre-treatment ultrasound evaluation of the breast and axilla was the same as that of the breast only.[8] Therefore, only the cost of ultrasound-guided needle sampling of the axilla was included for the costing model, taken from National Cost Collection for the NHS.[18]

Sentinel lymph node biopsy (SLNB) costs

SLNB is the preferred technique for staging the axilla for early invasive BC if no evidence of lymph node involvement on ultrasound or a negative ultrasound-guided needle biopsy.[28] Based on a BC costing study in England,[23] 87% , 68%, and 38% of stage 1, stage 2, and stage

© 2025 Wei X et al. *JAMA Oncology*.

3 BC were assumed to undergo this procedure. The SLNB cost was obtained from National Cost Collection for the NHS,[18] including the sentinel lymph node scan and unilateral intermediate breast procedure.

Axillary lymph node dissection (ALND) costs

ALND should be offered to women with positive axillary lymph node BC.[28] Cost of ALND is assumed to be 25% of the cost of breast surgery according to NICE BC guideline development group recommendation.[14]

Breast surgery costs

Breast surgery include breast conserving surgery and mastectomy. All women with non-invasive BC were assumed to undergo breast conserving surgery. Based on a costing study in England, 78%, 52%, and 33% of stage 1, stage 2, and stage 3 BC patients undergo breast conserving surgery, while 16%, 35%, and 67% undergo mastectomy (with/without reconstruction).[23] All stage 4 BC patients would undergo mastectomy. Approximately 23.3% of BC patients in the UK chose reconstruction following mastectomy.[24] The minor and major complication rate was reported to be 19.5% and 2.0% following mastectomy alone, and 24.5% and 4.1% following mastectomy and reconstruction.[25] The breast surgery costs were derived from National Cost Collection for the NHS,[18] using the procedure cost for Malignant Breast Disorders with Interventions, with CC Score 7+ (HRG code JA12D) and for Unilateral Delayed Pedicled Myocutaneous Breast Reconstruction (HRG code JA30Z). The minor or major complications costs were taken from literature.[20]

Chemotherapy and radiotherapy costs

Adjuvant therapy is offered to women with invasive BC who are not at low-risk as per NICE BC guidelines.[26,28] The costs of chemotherapy include planning, administration, first-line, second-line, third-line (where applicable) therapy, and related toxicity management, taken

© 2025 Wei X et al. *JAMA Oncology*.

from NICE advanced BC guideline.[26] 9%, 24%, 44%, and 100% of stage 1, stage 2, stage 3 BC, and stage 4 BC were assumed to receive chemotherapy.[23] Radiotherapy is offered at a treatment centre 5 days a week for 3 weeks, using external beam radiotherapy giving 40 Gy in 15 fractions.[28] 70%, 61%, and 83% of stage 1, stage 2, and stage 3 BC would receive radiotherapy.[23] Costs of planning and radiotherapy were taken from National Cost Collection for the NHS.[18]

Endocrine therapy costs

Women with ER-positive BC would receive endocrine therapy, with tamoxifen 20mg daily (premenopausal) or anastrozole 1mg daily (postmenopausal) according to NICE BC diagnosis and treatment guidelines.[26,28] The duration of endocrine therapy was assumed to be 5 years. The unit cost of tamoxifen and anastrozole was obtained from BNF,[21] and the ER test cost was taken from a local NHS trust and included for all invasive cancers.[29]

Biological therapy costs

Women with HER2-positive invasive BC are eligible for adjuvant trastuzumab therapy, given at 3-week intervals for 1 year or until recurrence.[28] 10% of the eligible patients were assumed to be intolerant of trastuzumab because of the risk of adverse events.[8] For patients suitable for trastuzumab treatment, 80% would receive the therapy.[8] It was assumed that 80% patients taking trastuzumab would experience disease progression outside the central nervous system, and 50% would continue taking trastuzumab.[8] The HER-2 test cost was taken from a local NHS trust and included for all invasive cancers.[29] The trastuzumab costs including administration and cardiac monitoring were £19,770 per patient, taken from NICE BC costing report.[8]

Bisphosphonate costs

© 2025 Wei X et al. *JAMA Oncology*.

Bisphosphonates should be considered for patients newly diagnosed with bone metastases to prevent skeletal-related events and reduce pain.[26] 74% advanced BC patients were assumed to develop bone metastases, and 87% of those with bone metastases were offered bisphosphonates.[8,27] The commonly used bisphosphonates included oral sodium clodronate, oral ibandronic acid, intravenous zoledronic acid, and intravenous pamidronate disodium,[8,28] and the proportions of patients receiving these drugs were 20%, 30%, 25% and 25%, respectively.[8] The annual drug costs (including administration costs) were £2,348, £3,333, £4,206, and £3,734, respectively, taken from NICE BC costing report.[8] The bisphosphonate treatment duration was assumed to be 2 years, which was based on the life expectancy of advanced BC with bone metastases.[30]

Follow up, recurrence, and terminal care costs

After active BC treatment, patients were assumed to receive clinical follow-up every four months in the first two years, every six months from the third to the fifth year, and every year from the sixth to the twentieth year. They were also offered annual mammography surveillance for twenty years. The cost of clinical follow-up was obtained from National Cost Collection for the NHS.[18] The recurrence costs were the sum of endocrine therapy, biological therapy, bone metastases treatment, and follow up costs for each BC stage. The terminal care costs for BC in the last year prior to death were derived from a UK study by Round et al.[31]

© 2025 Wei X et al. *JAMA Oncology*.

eMethods 3. Survival of breast cancer and impact of interventions

In our model, women were considered long-term survivors if they were alive without recurrence 20 years after diagnosis. Long-term cancer survivors were assumed to have the same probability of death as the general population. Estimates of BC survival for women at increased lifetime BC risk under screening are limited in the UK. The most recently available data are from the Manchester study of family history risk and prevention clinic, with 10-year survival of 90.76% (95%CI: 84.66%, 94.52%) for women with ≥17% lifetime BC risk under risk-stratified BC screening.[6] Due to the small sample size (N=254), they did not report the stage-specific survival for women at increased lifetime BC risk.

We therefore used the average survival across all stages and the stage distribution of women with 17%–30% and ≥30% lifetime BC risk under screening to estimate stage-specific 20-year survival. The key assumption was that the relative hazard across different stages for general population survival could be generalized to at-risk populations. First, we obtained the most recent 5-year survival estimates in general population for England from Cancer Research UK, which were 98.2%, 89.5%, 72.2%, and 26.6% for stage 1-4 BC.[16] These were be based on tumors diagnosed more recently than for the 10-year survival estimates. Assuming an exponential distribution of time to death, the hazard rate for stage 1–4 BC in general population was calculated using the following formula:

$$\text{Hazard rate} = \frac{\ln(5-year\ survival)}{-5}$$

This gives the hazard rate of 0.0036, 0.0222, 0.0651, and 0.2649 for stage 1–4 BC in general population respectively. The hazard ratio for stage 2, stage 3, stage 4 BC relative to stage 1 BC was 6.11, 17.93, and 72.91, respectively. These hazard ratios were generalized to women with 17%–30% lifetime risk and women with ≥30% lifetime risk under screening.

© 2025 Wei X et al. *JAMA Oncology*.

The 10-year survival for women with ≥17% lifetime BC risk under risk-stratified BC screening was 90.76% (95%CI: 84.66%, 94.52%).[6] This would give a weighted average hazard rate of 0.0097 across all stages. Using the stage distribution for women with 17–30% lifetime risk under screening (57.45%, 36.40%, 2.36%, and 3.80% for stage 1–4 BC) and the hazard ratios for stage 2–4 BC relative to stage 1 BC, the hazard rate for stage 1 BC would be 0.0016 for women with 17–30% lifetime BC risk under screening. The hazard rates of stage 2–4 BC were therefore 0.0099, 0.0290, and 0.1178, respectively. We can then estimate the 20-year survival of stage 1–4 BC using the following formula:

$$\text{Survival} = e^{-(time*hazard\ rate)}$$

The estimated 20-year survival of stage 1–4 BC were 96.82%, 82.08%, 56.00%, and 9.47% for women with 17–30% lifetime BC risk under screening, respectively.

Similarly, using the stage distribution for women with ≥30% lifetime risk under screening (67.95%, 30.17%, 0.68% and 1.20% for stages 1–4 BC) and the hazard ratios for stage 2–4 BC relative to stage 1 BC, the hazard rate for stage 1 BC would be 0.0028 for women with ≥30% lifetime BC risk under screening. The hazard rates of stage 2–4 BC were therefore 0.0168, 0.0493, and 0.2006, respectively. The estimated 20-year survival of stage 1–4 BC were 94.65%, 71.46%, 37.28%, and 1.81% for women with ≥30% lifetime BC risk under screening, respectively. The estimated 20-year survival of stage 1–4 BC were 92.99%, 64.16%, 27.17%, and 0.50% for general population without screening, respectively. The 20-year survival for DCIS was taken from literature[15], which was 96.70% (95%CI: 96.40%, 97.00%).

Due to lack of data, women who developed BC after RRM was assumed to have the same survival as those attending screening.

© 2025 Wei X et al. *JAMA Oncology*.

eMethods 4. Detailed utility value calculation

Quality-adjusted life-years (QALYs) are the recommended measure of health effects by NICE, which reflects both survival and health-related quality-of-life changes.[43] It equals time spent in a specific health state multiplied by the corresponding utility value. Utility value is an indicator of individual preference for a specific health state, where ‘1’ implies perfect health and ‘0’ implies death. Utility value is used for quality-of-life adjustment for different health states included in the model.

Utility values of RRM, medical prevention, and BC screening were derived from time trade-off (TTO) surveys among women with increased lifetime BC risks or general population.[33,34] Utility values of RRM, medical prevention, mammography screening, and MRI screening were 0.88, 0.95, 0.97, and 0.96, respectively.[33,34] The disutility of RRM were applied during the year of surgery. The disutility of BC screening attendance was applied for 1 week, and a disutility of 0.105 from a visual analogue scale (VAS) survey was applied for false positive results for 5 weeks.[35,36] The disutility for medical prevention was assigned for the duration of treatment.

Utility values of different BC stages were taken from a National Institute for Health and Care Research (NIHR) health technology assessment conducted by Robertson et al.[37] They adjusted estimates from a systematic review by accounting for the decrement from chemotherapy, resulting in utility values of 0.712 for DCIS, 0.597 for stage 1 BC, 0.527 for stage 2 BC, 0.497 for stage 3 BC, and 0.394 for stage 4 BC. Utility values of recurrent and remittent BC were derived from the pooling of utilities from studies using health state descriptions or standard gamble by Cooper et al,[38] which was 0.450 and 0.810, respectively. Utility of terminal BC (0.160) was derived from a literature review on utility values of BC related health states.[39]

© 2025 Wei X et al. *JAMA Oncology*.

All health state utility values were age-adjusted using multiplicative method,[43,44] which combined age-specific utilities in the 'healthy' state with utilities in all other health states, to account for the decline in utility due to aging. The mean population norms of utility values were taken from the Health Survey for England, where EQ-5D-3L questionnaire was used.[32] For example, the utility value for RRM was 0.88, and the utility value for the "healthy" state ranged from 0.913 to 0.692 for women aged 30 to 80 years. Applying the multiplicative method, this results in utility values for RRM ranging from 0.803 to 0.609 for women aged 30 to 80 years.

© 2025 Wei X et al. *JAMA Oncology*.

eMethods 5. Model validation

Validation efforts of the model were performed through a process of face, technical, and cross validity.[45,46]

Face validity: The model provides adequate representations of clinical reality. It incorporates all relevant elements associated with the dynamics of BC, ensuring that crucial aspects that could significantly impact the results are not overlooked. The model structure, key assumptions, and data sources were reviewed by BC screening and clinical experts, clinical geneticists, genetic epidemiologists, and health economists. These experts agreed that the model provides adequate pictures of the course of the disease and its clinical management in the UK. The results produced by the model are logically and intuitively consistent with the theorical basis of the disease and the expected impact of the interventions undertaken.

Technical validity/verification: To ensure the model behave as intended and are implemented correctly, systematic debugging was conducted. We performed structured "walk-throughs" of the model with BC clinical and health economic experts, to identify and rectify any errors or discrepancies in the coding or calculations. The models' technical functioning was also tested by extensive sensitivity analysis. The individual impact of each parameter on the results was examined in 1-way sensitivity analyses. Scenario analyses were conducted for different ages at RRM. The direction of changes in costs or health effects was consistent with expectations. Furthermore, the cancer incidence observed in the BC screening arm aligned with each assumed lifetime BC risk level, indicating that the model accurately reflects the natural progression of BC.

Cross validity: RRM has been found to be cost-effective compared with BC screening and medical prevention among *BRCA1*/*BRCA2*/*PALB2* PV carriers at varying surgery ages,[47,48] whose lifetime BC risks exceed 50%. Our model confirms the cost-effectiveness of RRM

© 2025 Wei X et al. *JAMA Oncology*.

among women with a lifetime BC risk over 35%. Unfortunately, there are no existing economic evaluations comparing RRM with BC screening for women with varying lifetime BC risks below 50%, limiting the cross validation of this model.

A limitation of the model validation is that the predictive validity or external validity of the model is difficult to test. Long-term real-world outcomes are currently lacking for women with an increased lifetime BC risk who are not PV carriers undergoing RRM. These validation efforts will only be possible when the real-world long-term empirical data become available.

© 2025 Wei X et al. *JAMA Oncology*.

eReferences

1. Rebbeck TR, Friebel T, Lynch HT, et al. Bilateral prophylactic mastectomy reduces breast cancer risk in BRCA1 and BRCA2 mutation carriers: the PROSE Study Group. *J Clin Oncol*. Mar 15 2004;22(6):1055-62. doi:10.1200/JCO.2004.04.188
2. Vreemann S, van Zelst JCM, Schlooz-Vries M, et al. The added value of mammography in different age-groups of women with and without BRCA mutation screened with breast MRI. *Breast Cancer Res*. Aug 3 2018;20(1):84. doi:10.1186/s13058-018-1019-6
3. Smith SG, Sestak I, Forster A, et al. Factors affecting uptake and adherence to breast cancer chemoprevention: a systematic review and meta-analysis. *Annals of Oncology*. 2016;27(4):575-590. doi:10.1093/annonc/mdv590
4. Cuzick J, Sestak I, Cawthorn S, Hamed H, Holli K, Howell A, Forbes JF. Tamoxifen for prevention of breast cancer: extended long-term follow-up of the IBIS-I breast cancer prevention trial. *Lancet Oncol*. Jan 2015;16(1):67-75. doi:10.1016/s1470-2045(14)71171-4
5. Cuzick J, Sestak I, Forbes JF, et al. Use of anastrozole for breast cancer prevention (IBIS-II): long-term results of a randomised controlled trial. *The Lancet*. 2020;395(10218):117-122. doi:10.1016/S0140-6736(19)32955-1
6. Evans DG, Howell SJ, Gandhi A, et al. Breast cancer incidence and early diagnosis in a family history risk and prevention clinic: 33-year experience in 14,311 women. *Breast Cancer Res Treat*. Oct 2021;189(3):677-687. doi:10.1007/s10549-021-06333-1
7. Brown LD, Cai TT, DasGupta A. Interval estimation for a binomial proportion. *Statistical science*. 2001;16(2):101-133.
8. National Institute for Health and Care Excellence. *National costing report: Early and locally advanced breast cancer/Advanced breast cancer*. National Institute for Health and Care Excellence; 2009. Accessed 1 July 2023.
9. Cancer Research UK. *Proportion Diagnosed by Stage (Breast cancer)*. 2023. Accessed 30 October 2023. https://crukcancerintelligence.shinyapps.io/EarlyDiagnosis/
10. Wapnir IL, Anderson SJ, Mamounas EP, et al. Prognosis after ipsilateral breast tumor recurrence and locoregional recurrences in five National Surgical Adjuvant Breast and Bowel Project node-positive adjuvant breast cancer trials. *J Clin Oncol*. May 1 2006;24(13):2028-37. doi:10.1200/jco.2005.04.3273
11. Anderson SJ, Wapnir I, Dignam JJ, et al. Prognosis after ipsilateral breast tumor recurrence and locoregional recurrences in patients treated by breast-conserving therapy in five National Surgical Adjuvant Breast and Bowel Project protocols of node-negative breast cancer. *J Clin Oncol*. May 20 2009;27(15):2466-73. doi:10.1200/jco.2008.19.8424
12. Gennari A, Conte P, Rosso R, Orlandini C, Bruzzi P. Survival of metastatic breast carcinoma patients over a 20-year period: a retrospective analysis based on individual patient data from six consecutive studies. *Cancer*. Oct 15 2005;104(8):1742-50. doi:10.1002/cncr.21359
13. Mavaddat N, Dorling L, Carvalho S, et al. Pathology of Tumors Associated With Pathogenic Germline Variants in 9 Breast Cancer Susceptibility Genes. *JAMA Oncol*. Mar 1 2022;8(3):e216744. doi:10.1001/jamaoncol.2021.6744
14. National Institute for Health and Care Excellence. *Early and locally advanced breast cancer: diagnosis and treatment*. National Collaborating Centre for Cancer, National Institute for Health and Care Excellence; 2009. Accessed 26 November 2022.
15. Narod SA, Iqbal J, Giannakeas V, Sopik V, Sun P. Breast Cancer Mortality After a Diagnosis of Ductal Carcinoma In Situ. *JAMA Oncol*. Oct 2015;1(7):888-96. doi:10.1001/jamaoncol.2015.2510

© 2025 Wei X et al. *JAMA Oncology*.

16. Cancer Research UK. *Survival and Incidence by Stage at Diagnosis (Breast cancer)*. 2023. Accessed 10 November 2023. https://crukcancerintelligence.shinyapps.io/EarlyDiagnosis/
17. Office for National Statistics. *National life tables: UK*. 2021. Accessed 19 August 2023. https://www.ons.gov.uk/peoplepopulationandcommunity/birthsdeathsandmarriages/lifeexpectancies/datasets/nationallifetablesunitedkingdomreferencetables
18. National Health Service. *National Cost Collection for the NHS*. 2022. Accessed 3 August 2023. https://www.england.nhs.uk/costing-in-the-nhs/national-cost-collection/#archive
19. Neuburger J, Macneill F, Jeevan R, van der Meulen JH, Cromwell DA. Trends in the use of bilateral mastectomy in England from 2002 to 2011: retrospective analysis of hospital episode statistics. *BMJ Open*. Aug 2013;3(8):e003179. doi:10.1136/bmjopen-2013-003179
20. Del Corral GA, Wes AM, Fischer JP, Serletti JM, Wu LC. Outcomes and Cost Analysis in High-Risk Patients Undergoing Simultaneous Free Flap Breast Reconstruction and Gynecologic Procedures. *Ann Plast Surg*. Nov 2015;75(5):534-8. doi:10.1097/sap.0000000000000156
21. British National Formulary. *British National Formulary*. BMJ Group and Pharmaceutical Press (Royal Pharmaceutical Society of Great Britain); 2021. Accessed 3 January 2023. https://bnf.nice.org.uk/
22. National Institute for Health and Care Excellence. *Costing report: Familial breast cancer*. National Institute for Health and Care Excellence; 2013. Accessed 27 November 2022.
23. Sun L, Cromwell D, Dodwell D, et al. Costs of Early Invasive Breast Cancer in England Using National Patient-Level Data. *Value in Health*. 2020;23(10):1316-1323. doi:10.1016/j.jval.2020.05.013
24. Jeevan R, Mennie J, Mohanna P, O'Donoghue J, Rainsbury R, Cromwell D. National trends and regional variation in immediate breast reconstruction rates. *Journal of British Surgery*. 2016;103(9):1147-1156. doi:10.1002/bjs.10161
25. Miller ME, Czechura T, Martz B, et al. Operative risks associated with contralateral prophylactic mastectomy: a single institution experience. *Annals of surgical oncology*. 2013;20(13):4113-4120. doi:10.1245/s10434-013-3108-1
26. National Institute for Health and Care Excellence. *Advanced breast cancer: diagnosis and treatment (CG81)*. National Collaborating Centre for Cancer, National Institute for Health and Care Excellence; 2009. Accessed 26 November 2022. https://www.nice.org.uk/guidance/cg81
27. National Institute for Health and Care Excellence. *Denosumab for the prevention of skeletal-related events in adults with bone metastases from solid tumours*. National Institute for Health and Care Excellence; 2012. Accessed 11 November 2022. https://www.nice.org.uk/guidance/ta265/documents/bone-metastases-from-solid-tumours-denosumab-final-appraisal-determination-guidance2
28. National Institute for Health and Care Excellence. *Early and locally advanced breast cancer: diagnosis and management (NG101)*. National Collaborating Centre for Cancer, National Institute for Health and Care Excellence; 2018. Accessed 7 November 2022. https://www.nice.org.uk/guidance/ng101
29. Sun L, Brentnall A, Patel S, et al. A Cost-effectiveness Analysis of Multigene Testing for All Patients With Breast Cancer. *JAMA Oncol*. Oct 3 2019;5(12):1718-30. doi:10.1001/jamaoncol.2019.3323

© 2025 Wei X et al. *JAMA Oncology*.

30. Coleman RE. Skeletal complications of malignancy. *Cancer: Interdisciplinary International Journal of the American Cancer Society*. 1997;80(S8):1588-1594. doi:10.1002/(sici)1097-0142(19971015)80:8+<1588::aid-cncr9>3.3.co;2-z
31. Round J, Jones L, Morris S. Estimating the cost of caring for people with cancer at the end of life: A modelling study. *Palliat Med*. Dec 2015;29(10):899-907. doi:10.1177/0269216315595203
32. Szende A, Janssen B, Cabases J. *Self-Reported Population Health: An International Perspective based on EQ-5D*. Springer; 2014. Accessed 24 August 2023.
33. Grann VR, Jacobson JS, Sundararajan V, Albert SM, Troxel AB, Neugut AI. The quality of life associated with prophylactic treatments for women with BRCA1/2 mutations. *Cancer J Sci Am*. Sep-Oct 1999;5(5):283-92.
34. Grann VR, Patel P, Bharthuar A, et al. Breast cancer-related preferences among women with and without BRCA mutations. *Breast Cancer Res Treat*. Jan 2010;119(1):177-84. doi:10.1007/s10549-009-0373-6
35. Geuzinge HA, Obdeijn I-M, Rutgers EJ, et al. Cost-effectiveness of breast cancer screening with magnetic resonance imaging for women at familial risk. *JAMA oncology*. 2020;6(9):1381-1389. doi:10.1001/jamaoncol.2020.2922
36. De Haes J, de Koning HJ, van Oortmarssen GJ, van Agt HM, de Bruyn AE, van der Maas PJ. The impact of a breast cancer screening programme on quality‐adjusted life‐years. *International journal of cancer*. 1991;49(4):538-544. doi:10.1002/ijc.2910490411
37. Robertson C, Arcot Ragupathy SK, Boachie C, et al. The clinical effectiveness and cost-effectiveness of different surveillance mammography regimens after the treatment for primary breast cancer: systematic reviews registry database analyses and economic evaluation. *Health Technol Assess*. Sep 2011;15(34):v-vi, 1-322. doi:10.3310/hta15340
38. Cooper NJ, Abrams KR, Sutton AJ, Turner D, Lambert PC. A Bayesian approach to Markov modelling in cost-effectiveness analyses: application to taxane use in advanced breast cancer. *Journal of the Royal Statistical Society: Series A (Statistics in Society)*. 2003;166(3):389-405. doi:https://doi.org/10.1111/1467-985X.00283
39. Peasgood T, Ward SE, Brazier J. Health-state utility values in breast cancer. *Expert review of pharmacoeconomics & outcomes research*. 2010;10(5):553-566. doi:10.1586/erp.10.65
40. Karen J, Amanda B. *Unit Costs of Health and Social Care 2021*. Personal Social Services Research Unit, University of Kent, Canterbury; 2021. Accessed 3 November 2023. https://www.pssru.ac.uk/project-pages/unit-costs/unit-costs-of-health-and-social-care-2021/
41. Organisation for Economic Co-operation and Development. *Inflation (CPI)*. 2022. Accessed 10 August 2023. https://data.oecd.org/price/inflation-cpi.htm
42. Organisation for Economic Co-operation and Development. *Purchasing power parities (PPP)*. 2022. Accessed 10 August 2023. https://data.oecd.org/conversion/purchasing-power-parities-ppp.htm
43. National Institute for Health and Care Excellence. *NICE health technology evaluations: the manual*. Process and methods [PMG36] ed. National Institute for Health and Care Excellence; 2023. Accessed 20 May 2024. https://www.nice.org.uk/process/pmg36/chapter/introduction-to-health-technology-evaluation

© 2025 Wei X et al. *JAMA Oncology*.

44. Ara R, Wailoo A. Using Health State Utility Values in Models Exploring the Cost-Effectiveness of Health Technologies. *Value in Health*. 2012/09/01/ 2012;15(6):971-974. doi:https://doi.org/10.1016/j.jval.2012.05.003
45. Eddy DM, Hollingworth W, Caro JJ, Tsevat J, McDonald KM, Wong JB. Model transparency and validation: a report of the ISPOR-SMDM Modeling Good Research Practices Task Force-7. *Med Decis Making*. Sep-Oct 2012;32(5):733-43. doi:10.1177/0272989x12454579
46. Hammerschmidt T, Goertz A, Wagenpfeil S, Neiss A, Wutzler P, Banz K. Validation of health economic models: the example of EVITA. *Value Health*. Sep-Oct 2003;6(5):551-9. doi:10.1046/j.1524-4733.2003.65241.x
47. Wei X, Sun L, Slade E, et al. Cost-Effectiveness of Gene-Specific Prevention Strategies for Ovarian and Breast Cancer. *JAMA Netw Open*. Feb 5 2024;7(2):e2355324. doi:10.1001/jamanetworkopen.2023.55324
48. Wei X, Oxley S, Sideris M, et al. Cost-Effectiveness of Risk-Reducing Surgery for Breast and Ovarian Cancer Prevention: A Systematic Review. *Cancers*. 2022;14(24):6117. doi:10.3390/cancers14246117

© 2025 Wei X et al. *JAMA Oncology*.
