# Supplementary material for: Defining Lifetime Risk Thresholds for Breast Cancer Surgical Prevention
Source: JAMA Oncol. 2025 Jul 24;11(9):1072–82. doi: 10.1001/jamaoncol.2025.2203 (PMC12290908; doi:10.1001/jamaoncol.2025.2203)
Supplement: Supplement 2. — Data sharing statement [file jamaoncol-e252203-s002.pdf]

**Data Sharing Statement**

**Data**
**Data available:** Yes
**Data types:** Other (please specify)
**Additional Information:** The data used or analyzed during the current study are publicly available. Data generated from the analysis are presented. Any additional data needed can be made available on reasonable request to the corresponding author - Ranjit Manchanda , email- r.manchanda@qmul.ac.uk
**How to access data:** The data used or analyzed during the current study are publicly available. Data generated from the analysis are presented. Any additional data needed can be made available on reasonable request to the corresponding author - Ranjit Manchanda , email- r.manchanda@qmul.ac.uk
**When available:** With publication

**Supporting Documents**
**Document types:** None

**Additional Information**
**Who can access the data:** Researchers whose proposed use of the data has been approved
**Types of analyses:** Specified purpose
**Mechanisms of data availability:** After approval of a proposal, with a signed data access agreement
